# Editorial: Positive and Negative Psychosocial Outcomes of the “Dark” Personality Traits

**DOI:** 10.3389/fpsyg.2022.919304

**Published:** 2022-05-16

**Authors:** Naser Aghababaei, Erin M. Lefdahl-Davis, Agata Błachnio

**Affiliations:** ^1^Department of Behavioral Sciences, The Institute for Research and Development in the Humanities (SAMT), Tehran, Iran; ^2^Department of Graduate Counseling, Indiana Wesleyan University, Marion, IN, United States; ^3^Department of Emotion and Motivation Psychology, The John Paul II Catholic University of Lublin, Lublin, Poland

**Keywords:** personality, Dark Triad, psychopathy, narcissism, Machiavellianism

Dark personalities refer to socially aversive traits such as spitefulness, greed, sadism, narcissism, psychopathy, and Machiavellianism in the subclinical range. Dark personality traits have attracted an exponential increase of empirical attention in recent years (e.g., Fernández-del-Río et al., 2020; Lebuda et al., 2021; Michels and Schulze, 2021). Much of the research in the last decade has linked these dark traits to negative psychosocial outcomes. Nevertheless, the dark personalities vary along a continuum of wellbeing and adjustment, with some showing more positive associations with mental health and wellbeing than others. After a decade of research into the positive and negative outcomes of dark personality traits, there is a need for studies to examine the mediational mechanisms that may explain the relationship between the dark personality traits and those outcomes. This Research Topic presents a set of studies examining the psychosocial correlates of the dark personality traits and consists of six articles that enhance our understanding of the structure of those traits and their positive and negative outcomes in several aspects of life including wellbeing, work, and deviant behaviors.

In line with the prior research (e.g., Aghababaei and Błachnio, 2015), Joshanloo showed that Machiavellianism and psychopathy were negatively and narcissism was positively associated with wellbeing. He found that conceptions of happiness were the mediating pathways through which the Dark Triad traits of Machiavellianism, narcissism, and psychopathy influence wellbeing. The Dark Triad traits are also associated with endorsing certain values that have implications for conceptions of wellbeing. People with various levels of the Dark Triad traits gravitate toward certain notions of wellbeing that are *per se* beneficial or detrimental to wellbeing. People high on Machiavellianism and psychopathy value and pursue happiness, but they have doubts about the consequences of happiness and their levels of personal control over it. Furnham and Treglown found that Dark Triad correlated with higher neuroticism and lower levels of Tolerance of Ambiguity and Conscientiousness among working adults. Job engagement mediated between Dark Triad traits and success ratings.

Leniarska and Zajenkowski's findings emphasize the importance of self-views related to intelligence for those with high narcissistic admiration. They found that narcissistic admiration was correlated with a low level distress and a high level of self-assessed intelligence. Additionally, intelligence self-views mediated the relationship between admiration and distress. Schade et al. set out to elucidate the associations between the Dark Triad, emotional intelligence, empathy, and cyberbullying in a community sample. They found that emotional intelligence partly mediate the Dark Triad associations with cyberbullying. And emotional intelligence appeared to buffer effects of grandiose narcissism on cyberbullying.

Mayer et al. used Theodore Millon's evolutionary personality theory to explore the life of Ferdinand Karl Piëch, an Austrian engineer and business executive who was the chairman of the executive board of the Volkswagen Group and the chairman of the supervisory board from 2002 to 2015. They suggested that although Piëch demonstrated the characteristics of non-conforming–antisocial, assertive-sadistic, confident-narcissistic, and paranoid-paraphrenic prototypes, the assertive-sadistic prototype is the best fit for his personality characteristics. Assertive-sadistic prototype is described as being opinionated, obstinate in holding to preconceptions, and exhibiting social intolerance and prejudice.

The Dark Triad have been found to negatively impact work behaviors including information sharing, reporting of unethical conduct, and mistreatment of colleagues and subordinates. However, research has found the Dark Triad can also be related to forms of organizational commitment which underpin positive work behaviors, including engaging in tasks and duties beyond those required. Professional commitment is a broader form of commitment that has been found to be significantly related to organizational commitment, sharing antecedents, and having similar outcomes. Professional commitment, that is the affective, normative, and continuance commitment toward one's profession or occupation, has the benefit of applying to individuals employed by organizations as well as those working for themselves or between jobs. Kaufmann et al. explored relationships between professional commitment, using previous research on the relationship between the Dark Triad traits and organizational commitment, as a basis for predictions, among Australian professionals. Their results showed that psychopathy had a negative association with affective commitment, whereas Machiavellianism was positively related to normative commitment, and narcissism was positively related to normative and continuance commitment.

These findings help expand our understanding of dark personality traits and their contingent impact on people' lives. As people from different cultures live their lives differently, practice different customs, have different child-rearing practices, and so on, it is also important to examine how culture exerts its influence on these traits. For instance, are there any cultural differences that may affect how people see a trait as “dark”? Are there any culture-specific dark personality traits? What are the culturally specific factors related to positive and negative outcomes of dark traits? How do these traits manifest themselves in various cultures and languages? These are fertile areas for future research.

## Author Contributions

All authors listed have made a substantial, direct, and intellectual contribution to the work and approved it for publication.

## Conflict of Interest

The authors declare that the research was conducted in the absence of any commercial or financial relationships that could be construed as a potential conflict of interest.

## Publisher's Note

All claims expressed in this article are solely those of the authors and do not necessarily represent those of their affiliated organizations, or those of the publisher, the editors and the reviewers. Any product that may be evaluated in this article, or claim that may be made by its manufacturer, is not guaranteed or endorsed by the publisher.
